# Between colonial medicine and global health: protein malnutrition and UNICEF milk in the Belgian Congo

**DOI:** 10.1017/mdh.2021.28

**Published:** 2021-10

**Authors:** Samuël Coghe

**Affiliations:** Global History, History Department, Freie Universität Berlin, Berlin, Germany

**Keywords:** Kwashiorkor, Colonial medicine, International health, Belgian Congo, FORÉAMI, Protein malnutrition

## Abstract

During the last decades of colonial rule, Belgian colonial authorities, health agencies and researchers intensely engaged with kwashiorkor, a severe syndrome that was deemed widespread among young children in some parts of the Belgian Congo and Ruanda-Urundi and chiefly attributed to protein malnutrition. To fight kwashiorkor, the Belgian government, in the early 1950s, set up a joint milk distribution campaign with the United Nations International Children’s Emergency Fund, Food and Agriculture Organization and World Health Organization, the first of its kind in colonial Africa. Placing this campaign in the context of mounting international and inter-imperial concern about kwashiorkor and other nutritional problems in Africa and across the globe, this article explores its rationales, mechanisms and consequences, and in particular, how the campaign was shaped and publicised by FORÉAMI, one of the main health providers on the ground. It not only contributes to the history of European colonial medicine and nutritional policies, but also opens new perspectives on international health collaboration during late colonialism. It argues that Belgian authorities were wary of international interference in colonial policies, but that especially FORÉAMI also viewed and used the campaign as an opportunity to display its ‘mastery’ in rural and infant healthcare and control the narrative on Belgium’s colonial medicine.

## Introduction

On 20th of April 1952, the steamer Alex Van Opstal left the port of Antwerp, bound for Matadi in the Belgian Congo. It carried 58.5 tonnes of skimmed milk powder bought in the Netherlands and paid for by the United Nations International Children’s Emergency Fund (UNICEF). Over the next 2 years, more than a dozen shipments followed, bringing 462 tonnes of skimmed and 104 tonnes of full milk powder to the Belgian Congo and Ruanda-Urundi in UNICEF’s very first campaign in sub-Saharan Africa.^1^

Lasting from 1952 to 1954, the campaign was a joint venture between the Belgian colonial government and UNICEF, with two other UN special agencies, the Food and Agriculture Organization (FAO) and the World Health Organization (WHO) as technical advisors. Its aim was to tackle kwashiorkor, an often-deadly health condition in young children that had been ‘discovered’ in the inter-war years and become a major concern of colonial governments in sub-Saharan Africa after the Second World War. Nowadays, kwashiorkor is considered a form of severe acute malnutrition, the most serious and fatal form of childhood malnutrition, and, although not yet completely understood, is usually attributed to the complex interplay between insufficient calorific and protein intake.^2^ In the 1950s, however, kwashiorkor was mostly defined as a ‘simple’ protein deficiency, to be cured and prevented by enriching local diets with protein-rich milk. While UNICEF provided the dried milk and covered the transport costs to Léopoldville (now Kinshasa) and Usumbura (now Bujumbura), the Belgian authorities distributed the milk to rural African populations in the regions that appeared most affected: the Kwango district and the Kasai province in the Belgian Congo, and the mandated territories of Ruanda-Urundi.

This article explores the rationales, mechanisms, tensions and consequences of this health campaign against the backdrop of global efforts to fight under- and malnutrition after the Second World War, particularly in what is now often called the Global South. By focusing on Belgian Africa and UNICEF, it adds to the expanding literature in this field, which has mostly dealt with other continents and actors, such as the US programmes of food distribution and technical assistance during the Cold War or the global nutrition programmes undertaken by FAO and WHO.^3^ It particularly engages with newer literature on kwashiorkor and protein malnutrition, which until recently have been neglected by medical historians of Africa.^4^ The article shows how, beyond the British and French colonies (mainly Uganda, Ghana and Senegal) analysed by Jennifer Tappan, John Nott and Lola Wilhelm, the Belgian territories in Africa also became important centres of kwashiorkor research in the late-colonial era.^5^ Moreover, the milk campaign differed from anti-kwashiorkor efforts in other parts of Africa in the early 1950s, as it was organised with and supervised by UN agencies and took a more preventative shape.^6^

Analysing the joint Belgian-UNICEF/FAO/WHO campaign also adds to the history of ‘global health’ in Africa. This somewhat elusive and contested term can broadly be defined as ‘efforts to improve the health of peoples living in countries that used to be called underdeveloped, or Third World, and are now known as low-income countries’ by actors based mainly outside these countries such as international health organisations, religious and/or private philanthropists and private–public partnerships, often in collaboration with (post)colonial states.^7^ Global health, a term mainly used for the period after 1990, is often said to have its roots in early twentieth-century colonial health campaigns and the ‘international health’ interventions that started in the early twentieth century and greatly expanded after the Second World War with the foundation of the WHO and other international health agencies.^8^

Only very recently have scholars begun to tease out the tensions between colonial states and international health organisations in late-colonial Africa, thereby also attending to inter-imperial forms of health collaboration, which became more strongly institutionalised after 1945.^9^ This article analyses how these tensions played out in the Belgian case, arguing that, as has been shown for the French and Portuguese, Belgian government officials feared the international interference and loss of sovereignty that international health campaigns might entail. However, they were not only financially motivated, as Pearson has argued for the French in West Africa, but saw and used collaboration with UN agencies as an opportunity to show their mastery of health matters, thus enhancing national prestige and legitimising colonial rule.^10^

What emerges here is not so much the ‘nervous’ side of the Belgian colonial state, concerned with security and surveillance and prone to violence, but a colonial state confident of the international appeal of its ‘biopolitical’ healthcare programmes.^11^ Belgian officials, I argue, managed to shape and publicise this campaign to their advantage, because it intersected with and was grafted upon long-standing practices of infant and rural healthcare as well as quickly expanding nutritional research in the Belgian Congo and Ruanda-Urundi.^12^ Structures and experiences in these domains converged in the western part of the Kwango district, where kwashiorkor was deemed to be particularly common and which was under the tight medical control of the *Fonds Reine Élisabeth pour l’Assistance Médicale aux Indigènes* (FORÉAMI).

This article attends to the pivotal role FORÉAMI played in this campaign. Founded in 1930, FORÉAMI was a powerful parastatal medical organisation. Like the AMI programme in neighbouring Angola, from which FORÉAMI’s founding father and director of the regular Belgian health services Giovanni Trolli drew much inspiration, it was financed through a special fund and operated as an autonomous entity parallel to the regular health services, having its own direction, personnel and budget. Though rooted in fighting sleeping sickness, it embraced a broader public health agenda. While mobile teams regularly canvassed the territory, divided in subsectors, to detect and treat a broad range of (endemic) diseases and take detailed censuses, FORÉAMI also set up a large network of rural dispensaries and, in collaboration with missionaries, infant and maternal welfare programmes.^13^

When, in 1935, FORÉAMI began to move its activities from the Lower Congo district to the Kwango district further east, FORÉAMI doctors found sleeping sickness and other endemic diseases less threatening than predicted.^14^ Yet they were struck by the chronic under- and malnutrition in most of the Kwango subsectors and the acute syndrome later called kwashiorkor. They held these nutritional conditions responsible for exceptionally high mortality and, together with a growing rural exodus, reduced demographic growth.^15^ Accounting for more than half of the official kwashiorkor cases in the Belgian Congo, the FORÉAMI Kwango sector, with its population of around 700 000 people, became the arena of sustained nutritional research and one of the hotspots of the Belgian-UNICEF milk distribution campaign after the Second World War.^16^

Letters, reports and nutritional studies produced (and in part published) by FORÉAMI doctors are crucial sources for this article. They are read in conversation with the records produced by the health, agricultural and administrative services in the Belgian Congo and senior officials in the Colonial and Foreign Ministries in Brussels, all held by the *Archives Africaines/Afrika-Archief* of the Belgian Ministry of Foreign Affairs and now gradually being transferred to the Belgian National Archives (*Algemeen Rijksarchief*). Taken together, these records offer very rich documentation on kwashiorkor research in the Belgian Congo, the campaign in Kwango and Belgium’s connections with international and inter-imperial health organisations. To assess the perspective of international organisations, I have also used WHO publications and a few selected sources from the WHO archives in Geneva and UNICEF archives in New York. All these sources, however, are largely silent on the viewpoints of the children and mothers targeted by anti-kwashiorkor measures, and do not allow to write a social history.

## The colonial ‘discovery’ of kwashiorkor

Kwashiorkor may have been long known by Africans, but European doctors only began to notice it after the First World War and particularly from the 1930s. In various parts of the continent, they described a severe syndrome that caused oedema, diarrhoea, discolouration of hair and skin, sores and other skin lesions, mainly in young children after weaning. Unless treated, in most cases, it was fatal. However, partly because they often had limited knowledge of similar cases elsewhere, doctors did not think about these conditions as a single disease nor did they agree upon a single aetiology.^17^ Cicely Williams, a paediatrician working in the British Gold Coast colony (now Ghana), was, in the mid-1930s, the first to attribute these symptoms to severe protein malnutrition. The name she used for the syndrome, kwashiorkor, meaning ‘the disease the deposed baby gets when the next one is born’ in the local Ga language, was later retained in medical nomenclature.^18^

Williams’s definition of kwashiorkor as a protein deficiency was not based on laboratory analysis of protein levels, but her observation of local diets and a differential diagnosis through which she excluded other nutritional diseases. Yet her deductions did not come out of thin air. In the inter-war years, colonial powers acknowledged that many Africans suffered from chronic undernutrition (due to a lack of calories) and/or malnutrition (due to a lack of vital nutrients such as (animal) proteins, fats, vitamins and minerals). These insights were premised on advances in nutritional science, which allowed better understanding of the function of particular nutritional elements (and redefinition of long-known diseases such as beri beri and pellagra as nutritional deficiencies), as well as better knowledge about the health and nutritional status of the Africans under colonial rule, due to tighter administrative control, anthropometric measurements and first nutritional field studies.^19^

In the early 1930s, one of these studies cemented the idea of a problematic protein gap in African diets. After comparing the diets, physical aspects and health conditions of the agricultural Kikuyu and the pastoral Maasai in Kenya, the British nutritionists John Boyd Orr (who would become the first FAO president) and J.L. Gilks concluded that animal proteins, which were much more prevalent in Maasai diets, had a very positive impact on bodily growth and health. Although this was only a preliminary study, their findings circulated widely and, as Cynthia Brantley has argued, were overinterpreted as the last word on the advantages of animal protein-rich diets. As such diets were rare in sub-Saharan Africa, the study suggested that protein malnutrition was widespread and that Africans needed more animal proteins in the form of milk or meat to stay healthy and (re)productive.^20^ Providing proteins to Africans, for instance, by promoting livestock husbandry, became an important aim of late-colonial development programmes.^21^

While Cicely Williams’s definition of kwashiorkor must be seen against the backdrop of these debates, there was no straight line between her work and the ‘protein consensus’ emerging around 1950. Although researchers in the 1930s and 1940s increasingly shifted their explanations from (parasitic) infections to some kind of nutritional disorder, many focused on the role of vitamins and minerals, which was the key field in nutrition science in the early twentieth century.^22^ Even doctors like Hugh Trowell, who would later play an important role in kwashiorkor research in Uganda, remained convinced that kwashiorkor was infantile pellagra (and hence caused by vitamin B deficiency) until well in the 1940s.^23^

This ontological and aetiological confusion was also tangible in the Belgian Congo. Here, the syndrome was first described by FORÉAMI doctors in the newly occupied Kwango sector in 1936–37, where it was well-known as *M’buaki* (‘red skin disease’) by locals. While van Daele readily identified protein malnutrition as its cause, most other doctors initially pointed at vitamin deficiencies, undernutrition or, as Trolli did, a combination of quantitatively insufficient and qualitatively unbalanced diets. None of them, however, referred to William’s work, which raises the question of the international visibility of her work at the time.^24^ In the 1940s, doctors in the neighbouring Kasai province linked a similar syndrome to ankylostomiasis, a parasitic disease, and iron deficiency.^25^ One of the reasons for this confusion, one of them later pointed out, was that the syndrome was usually found in rural areas lacking modern laboratories for medical research.^26^ It was only in the late 1940s and early 1950s, when kwashiorkor became the focus of more intense medical research, that cases across Africa and the world were connected and doctors agreed that this condition, with many different names and explanations, was one and the same syndrome caused mainly by a lack of protein.^27^ This consensus also resulted, as I will show, from research by new international health organisations such as WHO and FAO.

Recently, John Nott has argued that the British colonial administration actively contributed to misconstruing kwashiorkor as a form of malnutrition because that was easier to blame upon ecological conditions and ingrained African agricultural and nutritional customs than (politically sensitive) undernutrition.^28^ Evidence does not support this interpretation in the Belgian case. FORÉAMI doctors overtly analysed *M’buaki* against the conditions of both under- and malnutrition they encountered in the Kwango district. In the mid-1930s, they were the first to point out acute food scarcity *(disette*) in various parts of the district, particularly in the Feshi subsector where hundreds starved in 1936–37. They also acknowledged that undernutrition remained widespread in the 1940s, despite programmes of agricultural assistance, a problem that was also acknowledged by senior officials in the Colonial Ministry.^29^ Yet, just like Nott has argued for British Africa, doctors hardly discussed the potential role of colonialism in the emergence or increase of kwashiorkor, attributing it to seemingly permanent economic and cultural causes: poor soils, economic underdevelopment and general poverty, alongside the ‘innate laziness’ of ethnic groups in the region, lack of education, outdated agricultural methods and a preference for nutritiously poor staple crops such as cassava. Their approach was to improve agriculture and change local foodways rather than remedying the impoverishment, oppressive labour regimes and disrupted gender balances that, as social historians of Africans have shown, colonial rule and capitalism had brought about.^30^ Moreover, nutritional conditions probably still worsened during the Second World War, when forced labour and other forms of coercion were extended to support Belgium’s and the Allies’ ‘war effort’, thus causing severe hardships for the Congolese population.^31^

## From colonial malnutrition to international health

Kwashiorkor did not only occur in sub-Saharan Africa. In the early 1950s, and sometimes earlier, researchers acknowledged that it was identical to similar syndromes with different names that had long been observed in Central and South America, South-East Asia and, until the beginning of the twentieth century, also Europe. From a medical perspective, kwashiorkor was hence not framed as an ‘uniquely African tropical disease’, as Nott has contended, but as a paradigmatic disease of poverty in ‘underdeveloped’ countries, whose geography around 1950 was more or less congruent with the emerging ‘Third World’, now often referred to as the Global South.^32^ While its biomedical, social and agricultural dimensions attracted much research, (colonial) governments, often collaborating with specialised UN agencies, organised emergency campaigns with milk and other protein-rich foods and a range of agricultural development measures to cure and prevent the condition.^33^ If Africa became an important (though not the only) focus of kwashiorkor research and campaigns after the Second World War, that was due partly to the apparently higher incidence of severe cases (itself a consequence of comparatively poor nutritional conditions and healthcare systems)^34^ and partly, perhaps even mainly, to the particular configurations of late-colonial rule in Africa. The Belgian Congo illustrates how nutrition and child health came to sit at the core of late-colonial ideologies and practices of health and development, and how, after 1945, colonial health interventions became entangled with inter-imperial and (regional branches of) international organisations.

With epidemic and endemic diseases increasingly under control, colonial governments in post-1945 Africa now considered under- and malnutrition as a major health problem and obstacle to development.^35^ At the same time, the meaning and urgency of development was changing, as it was increasingly defined as a combination of economic progress and social welfare and viewed as an antidote to centrifugal anticolonial tendencies in a decolonising world. European governments committed, at least on paper, to developing their colonies and raising the standard of living for local populations in a more substantial way through investment funds and long-term development programmes.^36^ In 1947, the Belgian government created the *Fonds du Bien-Être Indigène* (FBEI, ‘Native Welfare Fund’) to improve public health in rural areas, mainly through investing in health infrastructure.^37^ A few years later, the Belgian state launched its first 10-year development programmes for the Belgian Congo and Ruanda-Urundi. Besides important investments in the health sector, the *Plan Décennal* (1949–59) for the Congo included a host of agricultural development measures aiming to boost the export of colonial cash crops, and improve food security for colonial populations by producing more calories and protein. Framing nutrition as an agricultural problem, its goal was to more than double the production of protein-rich foodstuffs such as groundnuts, meat, fish and milk by 1959.^38^

In this context, nutritional research was institutionalised in Belgian Central Africa. In 1947, the Colonial Ministry in Brussels nominated E.-L. Adriaens, the chief chemist of the nutritional laboratory in Tervuren, to conduct a study that, for the first time, would analyse the biochemical constitution and social meaning of daily diets in the Belgian Congo. In 1948, Adriaens conducted his initial surveys in the Kwango Sector of the FORÉAMI, who had offered its medical infrastructure and the collaboration of one of its doctors, thus underscoring its ambition in the field of nutritional research.^39^ Officially, however, Adriaens’s mission was under the guidance of the newly founded *Institut pour la Recherche Scientifique en Afrique Centrale* (IRSAC) and its first director, Louis Van den Berghe, Professor at the Antwerp School of Tropical Medicine. With its headquarters in Lwiro (Kivu Province), and various other research centres across Congo and Ruanda-Urundi, IRSAC became the most important research institution for the natural and social sciences in Belgian Africa. Nutritional research was high on its agenda. Led by Professor Bigwood, IRSAC’s nutritional section was financially supported by the FBEI and collaborated with the main agricultural station of the *Institut National pour l’Étude Agronomique du Congo Belge* (INÉAC) in Yangambi as well as private and state medical laboratories.^40^ Throughout the 1950s, IRSAC nutritionists such as Demaeyer and Vanderborght conducted important research on kwashiorkor.^41^ FORÉAMI also contributed to the further institutionalisation of nutritional research in the Belgian colonies. In 1953, it established a small, though quite productive nutritional laboratory in Feshi that played an important role in kwashiorkor research.

To understand why kwashiorkor gained so much attention, it is important to note that, as a nutritional condition affecting mainly young children and pregnant or nursing women, kwashiorkor research and interventionism intersected with the mounting importance of child and maternal health in late-colonial Africa. While this development can be observed for all colonial powers, it was particularly strong and early in the Belgian colonies, as Nancy Rose Hunt has amply demonstrated. Driven by demographic anxieties, after the First World War the Belgians devised a broad set of policies to increase fertility and diminish infant and maternal mortality in their colonial territories, ranging from pro-natalist campaigns against polygamy, birth spacing and venereal diseases to the establishment of midwife schools, maternities and infant welfare centres.^42^ Interventions increased under the welfarist agendas of post-Second War development programmes. By the 1950s, an ‘array of public and private organisations was involved in maternal and infant health’ (including FORÉAMI) and the centres providing prenatal consultations, medicalised childbirth and infant consultation programmes were arguably unrivalled in ‘number and importance’ in colonial sub-Saharan Africa.^43^

The importance that Belgian and other colonial governments now attached to nutritional problems was also reflected in (and further amplified by) the fact that they turned it into a key issue of inter-imperial exchange and collaboration within the Commission for Technical Co-operation in Africa South of the Sahara (CCTA). Founded in 1949/50 after several multilateral meetings and conferences, the CCTA promoted information exchange and mutual collaboration between its member states (initially the colonial powers of the United Kingdom, France, Belgium, Portugal, South Africa and Southern Rhodesia; independent African states started to join in 1957) on a range of technical and social issues such as human and animal African trypanosomiasis, medical cooperation, soil erosion and rural welfare.^44^ Between 1949 and 1961, the CCTA organised four major conferences on nutritional problems in Africa (Dschang 1949; Fajara 1952; Luanda 1956 and Douala 1961) and, from 1954, regular meetings of a select group of nutritional experts. Until decolonisation in 1960, Belgian representatives eagerly participated in these meetings during which the incidence, treatment and prevention of kwashiorkor was prominently discussed.^45^

It has often been argued that, by showcasing technical expertise and action in the same fields of expertise, the CCTA was intended to keep specialised UN agencies such as WHO (including its regional branch AFRO), FAO or ILO and their reputedly anti-colonial agendas out of Africa. France, Belgium and Portugal in particular have been said to promote inter-imperial cooperation in Africa as a way to ward off international interference in their colonies.^46^ However, the CCTA did not have a budget to fund larger health projects in the colonies and, by the late 1950s, even ‘sceptical’ colonial states such as Portugal made increasing use of AFRO’s budgets for technical assistance.^47^ In the case of kwashiorkor, colonial attempts to use the CCTA against international health organisations backfired from the outset. In ways similar to what McVety has described for the inter-colonial conference on rinderpest in 1948, the CCTA was not able to prevent the participation of FAO and WHO officials at their nutritional conferences, as the diplomatic costs would have been too high.^48^ Certainly, they only received the status of ‘observers’, but, as I will show, their presence at the conferences in Dschang (1949) and Fajara (1952) contributed to propelling kwashiorkor onto the agenda of these international UN agencies.

That international agencies were at all interested in participating resulted from the global importance that mal- and undernutrition had gained. Between the two world wars, international agencies such as the League of Nations, the International Labour Office and the Rockefeller Foundation had already fostered research into the agricultural, economic and medical dimensions of under- and malnutrition.^49^ Yet it was the hunger crisis triggered by the Second World War in Europe and Asia that turned the global production and distribution of basic foodstuffs into a crucial concern for the Allies (in their plans for a post-war peaceful world) and for various new international agencies in the United Nations orbit, such as the United Nations Relief and Rehabilitation Administration (UNRRA) and, after its creation in 1945, the FAO. Framing ‘freedom from want’ as a basic human right, they initiated emergency food aid campaigns in Southern and Eastern Europe and East Asia and launched programmes of technical assistance to enhance local food production.^50^

Colonial sub-Saharan Africa was initially left out of these campaigns, but around 1950, both FAO and WHO started shifting their interest to nutritional and other health problems in Africa, particularly kwashiorkor.^51^ In 1949, they formed a Joint Expert Committee on Nutrition to explore common ground. Although they looked at nutrition from different angles – the FAO ‘in relation to the production, distribution, and consumption of food’ and the WHO ‘in relation to the maintenance of health and the prevention of disease’^52^ – their nutrition programmes bore so many overlaps that both agencies considered closer collaboration advisable. Already at its first meeting, the Committee expressed great interest in kwashiorkor, characterised as ‘one of the most widespread’, yet still ‘ill-defined’ ‘nutritional disorders in tropical and subtropical areas’.^53^ This did not happen by accident: only 3 weeks earlier, the directors of FAO and WHO’s nutritional divisions, W.R. Akroyd and F. Clements, who acted as secretaries to the Joint Expert Committee, had both attended the CCTA conference in Dschang.^54^ Upon the Committee’s recommendation, a joint research mission led by nutritionists John Fleming Brock from the University of Cape Town and Marcel Autret from FAO was sent to tropical Africa. For months, they studied the incidence and clinical features of the disorder, the food habits of the populations concerned, effective treatments, preventative measures and ongoing research efforts in various British, French and Belgian colonies. Their report was presented at the Committee’s second meeting in Rome in April 1951 and became the authoritative study in the field.^55^ It provided the decisive impetus for international health campaigns against kwashiorkor with protein-rich foods in what was later termed the ‘protein decade’.^56^

## Negotiating a UNICEF milk campaign in the Belgian Congo and Ruanda-Urundi

Brock and Autret could not unravel all the details of kwashiorkor’s aetiology. Yet, on the basis of epidemiological data, nutritional analyses and patient histories, they confirmed the by then dominant hypothesis that kwashiorkor was intimately related to protein deficiency; that is, the lack of certain nutritional elements normally contained in animal and high-quality vegetable proteins.^57^ The long-term solution they proposed was to increase young children’s consumption of animal (or, alternatively, vegetable) proteins via agricultural and educational measures. Such measures would also relieve general food shortages during the so-called ‘hungry months’ before a new harvest. As it would take time to change the nutritional landscape in tropical Africa, they recommended that short-term action should focus on the distribution of milk powder to ‘hospitals and maternity and child-welfare centres’ in affected regions, as a high protein/low fat diet based on skimmed milk had proved efficient in treating early cases.^58^ Governments, the report added, could get help from UNICEF in this matter.^59^ Reiterated by the Joint Expert Committee on Nutrition during its second meeting, this offer paved the way for UNICEF’s milk campaigns in Central Africa.^60^

By the early 1950s, UNICEF’s experience in supplying dried milk to the world was unrivalled. Founded in the wake of the Second World War to provide emergency aid to Europe’s undernourished children, UNICEF set up huge milk distribution campaigns in Europe and later in Latin America, the Middle East and (South) East Asia. Alongside its investment in mass health campaigns for children and training programmes for health workers, milk distribution and conservation programmes constituted UNICEF’s core business. As Gillespie has argued, UNICEF’s focus on milk was driven not only by its nutritional value, but also by the cheap supply of surplus US milk that had shaped previous UNRRA programmes as well.^61^ In 1951, the mounting international attention on kwashiorkor dovetailed with UNICEF’s plans to expand into sub-Saharan Africa. In May 1951, UNICEF’s executive board approved a $2 million budget line for projects in Africa. While supplies and equipment for basic maternal and child welfare schemes, training programmes and mass health campaigns against malaria, tuberculosis and trachoma accounted for more than half of that money, the remaining $800 000 was reserved for supplementary feeding programmes ‘including, *inter alia,* milk conservation’.^62^

To get its African programme off the ground, UNICEF actively approached colonial governments. As a senior health official in the Belgian Colonial Ministry in Brussels later trenchantly observed in an internal note, ‘if the [Belgian] government decided to ask UNICEF for help that was by no means indispensable, that was upon pressing requests on the part of the UNICEF administration itself’.^63^ Back in 1951, however, UNICEF’s offer constituted a great opportunity for the Belgian colonial government to tackle kwashiorkor on an unprecedented scale. In a note to the Belgian delegation at the UN Economic and Social Council (ECOSOC), the body that governed the UN special agencies, the Inspector-General of the Health Services in the Belgian Colonial Ministry, Dr Duren, stated that the ‘medical authorities of the Belgian Congo and Ruanda-Urundi would […] very much like to benefit from UNICEF’s support in providing milk powder and other weaning foodstuff’. Belgian health authorities had substantially lowered infant mortality through malaria prophylaxis and the treatment of pulmonary diseases, he explained, but ‘were still struggling with the big problem of undernutrition that affected almost all Congolese children after their first year of life’. It caused deficiency syndromes such as kwashiorkor and raised children’s general susceptibility to infectious and parasitic diseases.^64^

Duren’s support proved crucial. After meeting with representatives of FAO, WHO and UNICEF in Geneva and getting favourable advice from other departments in the Colonial Ministry, he convinced the Minister of Colonies that UNICEF support was useful.^65^ In October 1951, the Belgian Foreign Ministry filed a demand to the UNICEF branch in Paris, responsible for Europe, the Middle East and Africa. It asked UNICEF to provide 342 tonnes of skimmed milk powder annually for children in the Belgian Congo and Ruanda-Urundi suffering from or threatened by kwashiorkor. In the Belgian Congo, this concerned about 20 000 children and pregnant or nursing women (1% of the population) in the Kwango district and Kasai province. In Ruanda-Urundi, kwashiorkor was considered less widespread and to mainly affect the Hutu majority, who possessed fewer cattle and had less access to milk products than the pastoral Tutsi. In addition, the Belgians also asked for 55 tonnes of full milk powder annually to improve the diet of 2 000 orphan babies under government care across the Belgian Congo and Ruanda-Urundi.^66^

In line with the official position of UNICEF, FAO and WHO, the Belgian government framed UNICEF’s support as a temporary measure to be gradually phased out as the local production of (animal) proteins scheduled in the 10-year-development programme progressed. Belgian’s development programme for the Congo promised to increase the production of protein-rich foods, especially of animal origin, through the extension and improvement of agriculture, fish farming and animal husbandry. By 1959, these measures should have multiplied the average intake of animal proteins from 2 to 7 g per day per person, and total protein consumption to 50 g. Belgian efforts to ensure the temporariness of milk distribution constituted an important precondition for UNICEF and its partners to approve the request, which eventually happened in April 1952.^67^

Even so, cooperation between the Belgian colonial government and an international agency such as UNICEF was not self-evident. Similar to what Pearson has argued for French colonial Africa after the Second World War, the Belgians feared that UNICEF and the other international agencies might interfere in colonial policies.^68^ Before the demand was filed, a senior official in the Colonial Ministry explicitly warned that ‘the Colony should not give up its independence and make sure that the conditions proposed by the donor do not allow him to interfere in how we want to distribute or use this milk’.^69^ The negotiations between UNICEF (assisted by WHO and FAO) and the Belgian government in early 1952 reflected these anxieties. Against UNICEF’s demand for a permanent presence of UNICEF officials, the Belgian Colonial Ministry acknowledged UNICEF’s right to control the execution of the programme, but only agreed to allow a few temporary visits to the sites of the programme by ‘accredited officers chosen in agreement with the government’.^70^

## Implementing the milk campaign: the case of the FORÉAMI Kwango sector

Between September 1952 and December 1954, various health organisms participated in the local execution of the joint Belgian-UNICEF milk campaign. While the ‘regular’ government health services were in charge of milk distribution in the Kasai province and the mandated territories of Ruanda-Urundi, the Kwango district was served by two parastatal health agencies, the Mission Médicale du Kwango (MMK) and the FORÉAMI. To distribute milk, they used the health infrastructures established by earlier (and ongoing) general health and infant welfare campaigns: state and mission hospitals, rural dispensaries, maternity clinics and infant welfare centres. Usually, it was European health workers in these institutions who cooked the milk ‘on the spot’ and distributed it in its reconstituted, liquid form on a daily or weekly basis.^71^

Beyond those suffering from acute kwashiorkor, the scheme preventively targeted all children between one and five, deemed most vulnerable to protein malnutrition; women in the last 2 months of pregnancy and breastfeeding mothers. Individual rations of skimmed milk roughly followed the recommendations elaborated in collaboration with FAO and WHO experts. They varied according to age and health, with hospitalised kwashiorkor patients receiving up to 100 g of milk powder daily as part of a curative treatment and ‘healthy’ children somewhere between 20 g (under 2 years) and 40 g (2–5 years) as a preventative measure. Pregnant and nursing women usually received about 40 g daily.^72^ In practice, volume and regularity of distribution also depended on how health organisms solved logistical problems and managed to convince potential beneficiaries of the scheme’s health benefits.

The manifold tensions of the milk campaign become clear when analysing its implementation in the FORÉAMI Kwango sector, which quickly became the centrepiece of the milk programme, receiving, as I will show, much attention and praise from both Belgian and international observers and an increasing share of UNICEF milk. There were several reasons for this. On the one hand, FORÉAMI could rely on a particularly dense network of 44 milk distribution centres, due to its long-standing medical occupation of the area and the additional support of missionaries.^73^ It also had exceptional experience in infant healthcare. At the eve of the campaign, about 50% of the children under three were reportedly enrolled in FORÉAMI’s infant welfare centres, with half of them showing up regularly – very high figures that still went up during the 1950s.^74^ On the other hand, FORÉAMI adopted innovative solutions to problems of logistics, staff and scientific control, and was also keen on publicising them.

Yet, even in the Kwango sector, reaching people and changing their diets was difficult. One of the main obstacles for the campaign was that locals were not used to drinking cow milk and many women were reluctant to drink it or feed it to their children. Certainly, some infant welfare centres in the Belgian Congo had, inspired by the so-called *gouttes de lait* (‘milk drops’) in France, Belgium or Great Britain, begun to distribute milk to indigent children and orphans in the 1920s, as in other African colonies.^75^ Yet these initiatives were usually confined to urban areas or major companies, since the dairy industry in the Belgian Congo (situated mainly around cities in Haut-Katanga, Ituri and northern Kivu) was limited and imported milk very expensive.^76^ The joint Belgian-UNICEF campaign was the first major attempt to introduce dairy milk to the diets of *rural* Congolese children and mothers.^77^ Resistance was partly inspired by local customs and beliefs, which European colonialists often dismissed as ‘food taboos’, but which were probably due to widespread lactose intolerance. Nowadays, about 95% of adults in the Democratic Republic of Congo suffer from lactose malabsorption, a genetically or environmentally induced condition that often emerges after weaning and is called lactose intolerance when causing symptoms such as diarrhoea, nausea or abdominal pain.^78^ This would explain the diarrhoea and nausea that, according to FORÉAMI reports, caused some children and women to withdraw from the milk programme. Pregnant women were particularly hard to convince, as some of them feared that supplementary milk would increase the weight of their babies and hence the risk of birth complications. Yet, most FORÉAMI agents were confident that the programme would change these attitudes. While they added sugar to the milk to meet adult women’s taste, they observed how propaganda efforts and visible health benefits caused many to comply, sometimes even enthusiastically.^79^

Another major problem was logistical. The use of fixed milk distribution points obliged women and children to walk long distances. Many potential beneficiaries did not attend regularly, particularly those who lived far from the fixed centres.^80^ To enhance the reach of the campaign, FORÉAMI set up an itinerant distribution scheme in one of the six Kwango subsectors that brought milk directly to the villages. Not coincidentally, this pilot project took place in the Feshi subsector, where, as mentioned earlier, under- and malnutrition had been endemic among the Basuku people for many years. Statistics even suggested that, in some parts of the Feshi subsector, kwashiorkor (broadly defined to include mild and precursor forms) affected almost half of the children between two and five.^81^ The mobile scheme was far more labour-intensive and expensive than fixed distribution centres. It was initially executed mainly by car by the Belgian sanitary agent Ysebaert and his wife, but reorganised several times in 1953 to make it more regular and effective. Eventually, itinerant milk distribution relied almost exclusively on African nurses and porters who prepared and delivered milk to more than 60 dispersed villages on predefined routes.^82^ In this way, some 3 000 Congolese children and women received UNICEF milk daily.^83^ Both in terms of costs and volume of milk powder distributed, this itinerant scheme was the centrepiece of FORÉAMI’s contribution to the joint Belgian-UNICEF milk campaign.

FORÉAMI itself was very quick to publicly announce the positive results of its work. In a report compiled after only 6 months and published in Brussels, most doctors and sanitary agents in the Kwango subsectors indicated that skimmed milk had diminished the mortality rates and hospitalisation time of kwashiorkor patients. If taken preventatively, skimmed milk also appeared to have a positive effect on general health, improving the health and stamina of pregnant women, the weight of newborns and the weight curve of infants.^84^ The most spectacular results came from the Feshi subsector. A study conducted in seven of the villages served by the itinerant scheme confirmed the general health benefits of skimmed milk and its protective effect against kwashiorkor. While there had been 70 mild cases at the start of the scheme, no new cases appeared during the campaign.^85^ Although the itinerant scheme was the most difficult and expensive to organise, the report concluded, it ‘responded best to the preventative and curative goals that we had committed to’.^86^

Subsequent studies confirmed the positive effects of the itinerant scheme. In order to monitor the milk campaign and conduct further nutritional studies in the region, in 1953 FORÉAMI had set up a nutritional laboratory in Feshi under the direction of Dr Holemans.^87^ After 15 months, Holemans presented further, biochemical proof of milk’s positive impact: beyond higher and more regular weight curves, which also stood out in an anthropometric study by sanitary agent Ysebaert, supplementary milk had led to better haemoglobin and serial protein rates among Feshi’s rural population, improved the quantity and composition of women’s breastmilk and reduced general mortality in children under three. It did not seem to have increased the weight of newborns, however, thus contradicting African women’s fears of heavy babies.^88^

From early on, FORÉAMI’s efforts also received much praise from the international agencies co-organising the milk programme. The joint UNICEF/WHO/FAO mission, which visited the whole programme in September to October 1952, was enchanted by the efforts of all Belgian health agencies, but especially FORÉAMI. The mission members particularly lauded the itinerant scheme as an ideal and unprecedented solution for rural regions with very high incidences of protein malnutrition, while also recommending some changes (such as the use of African staff) to further extend its reach.^89^ Their positive evaluation and the subsequent reports and studies by FORÉAMI, which ‘for the first time […] in such an environment and under conditions in the African backwoods’ offered scientific proof of the benefits of supplementary milk, were instrumental in convincing UNICEF to prolong the project. In 1954, it allocated a second lot of 120 tonnes of skimmed milk to the Belgian Congo, mainly destined for the Kwango district.^90^

For the UN agencies involved, the Belgian campaign was obviously a greater success than that in neighbouring French Equatorial Africa (AEF). In April 1952, UNICEF’s programme committee had, alongside the Belgian request, approved a similar French demand of supplementary milk for the AEF, a region that also suffered from protein malnutrition.^91^ The French started their campaign with a pilot project in Brazzaville and two rural districts in the colonies of Moyen-Congo and Oubangui-Chari. During their evaluation, however, the same UNICEF, FAO and WHO experts who had visited the Belgian Congo noted that the incidence of protein deficiency was too low in these regions for the milk campaign to make a real difference and be cost-effective. They urged the French to change location and method, but these ultimately decided to abandon the whole programme.^92^

However, FORÉAMI’s conclusions regarding milk’s preventative effects were not shared by other Belgian health organisms. While the medical department in Kasai province also acknowledged them, the MMK and the health services in Ruanda-Urundi were unconvinced and concluded that the milk should only be used to treat kwashiorkor in hospital. Preventative distribution, the MMK director maintained, ‘must be considered as waste unless the milk were transported by car into each village’, for which they had neither money nor personnel.^93^ FORÉAMI’s itinerant campaign was clearly not deemed replicable, or worth the effort, in other areas.

This negative stance was underwritten by barely hidden racist reservations about African auxiliaries. In April 1953, reacting to the recommendation from FAO/WHO/UNICEF mission members to use African staff for the itinerant scheme, the Governor-General of the Belgian Congo Léo Pétillon stressed the great dangers of leaving Africans in command.^94^ These echoed widespread anxieties vis-à-vis the ‘Africanisation’ of state services in colonial Africa. Many colonial officers assumed that Africans could not take responsibility without regular supervision, as they were not yet civilised enough to resist corruption and idleness, or to think for themselves. European doctors in the Belgian Congo repeatedly voiced similar reservations about the African nurses and midwives they educated, limiting their career paths and salaries and impeding the education of fully fledged Congolese doctors.^95^ Although, by 1952, FORÉAMI employed hundreds of African assistant-nurses, they were only gradually drawn into the itinerant scheme and, after their ‘initiation’, closely controlled by European staff.^96^

Belgian administrative authorities objected to preventative milk distribution in rural areas for other reasons as well. Governor-General Pétillon feared the social and financial consequences of creating new dietary habits among rural populations whose purchasing power was ‘forcibly reduced because of poor soils and low crop yields’. He disliked the idea of continuing a large preventative free milk campaign after 1954 at the expense of the Belgian state, as agreed with UNICEF, since this would put a huge strain on the colony’s budget.^97^ Though seeing the same problem, the governor of the Kasai Province was more optimistic. He wished to wait until the Belgian take-over of the campaign in 1955 to limit the free distribution of milk powder to curative treatments, confident that economic development and rising standards of living would by then allow many Africans to buy milk powder themselves.^98^

## Beyond milk? The campaign’s afterlife

After the end of UNICEF’s commitment, the milk campaign lost momentum. Bound by its engagement with UNICEF, FAO and WHO, the Belgian government continued the free distribution of skimmed milk powder to fight kwashiorkor for a few years. Yet budgets and ambitions were reduced. From the 2 million francs originally budgeted for 1955, for instance, only 1 million was eventually made available.^99^ Given the aforementioned reluctance of health and administrative authorities, the preventative use of milk was mainly confined to the FORÉAMI sector in the Kwango district, where the scheme continued until 1957.^100^

For Dr Holemans, the preventative value of skimmed milk remained uncontested. In 1957, he bragged that, due to FORÉAMI’s milk scheme, cases of severe final-stage kwashiorkor ‘that had been so frequent in this territory 6 years ago have virtually disappeared’.^101^ Indeed, between 1951 and 1957, overall case numbers (including mild and precursor cases) in the FORÉAMI sector had fallen from 6 405 to 2 887 and mortality declined from 212 to 120, with ‘only’ 16 victims in the Feshi hospital.^102^ To what extent this was due to preventative milk distribution is uncertain, however, since this trend was also observed in other parts of the Belgian Congo and Ruanda-Urundi. In hindsight, IRSAC nutritionists attributed the decline of severe cases to general socio-economic and hygienic improvements and earlier hospitalisation of malnourished children due to parents’ rising confidence in biomedical kwashiorkor treatment.^103^ Moreover, some FORÉAMI doctors grew increasingly critical of the itinerant scheme. Dr André, director of the Kwango sector, for instance, openly complained in 1957 that it was labour-intensive and expensive and that its results depended on how closely the African personnel were supervised, recommending that distribution should be limited to fixed centres.^104^

Concurrently, FORÉAMI increasingly turned to other protein-rich foods to tackle malnutrition. Right from the start of the campaign, FORÉAMI commissioned manufacturers in Belgium and Léopoldville to develop flour and biscuits enriched with milk powder, deemed easier to store and distribute. The flour was unpopular, but children in the Kwango district found the biscuits palatable. Production and transport were very expensive, however, and FORÉAMI abandoned this project.^105^ An alternative high-protein foodstuff was peanuts. Between July 1954 and October 1955, FORÉAMI distributed peanut milk and, for the older children and women, roasted peanuts in a small pilot area of five villages near Feshi.^106^ Compared to milk, peanuts were much cheaper, locally produced and already part of local diets. Furthermore, unlike soybeans, the taste was widely accepted by the local population.^107^ When the laboratory in Feshi evaluated the experiment, Holemans and Lambrechts found health benefits similar to those of skimmed milk among the 135 infants and 96 nursing mothers who had participated.^108^ Consequently, Dr André recommended that, in areas where malnutrition was still endemic, skimmed milk should be replaced with peanuts or protein-enriched flour.^109^

FORÉAMI’s experiments were part of an international quest in the 1950s and 1960s to find substitutes for milk, a product that was often not part of local diets, not easily available and, like other animal proteins, expensive ‘in terms of land, labour, materials and time’. With animal proteins only, it would be impossible to reduce kwashiorkor in large parts of the world, leading British kwashiorkor experts in Uganda wrote.^110^ The search for alternative plant proteins also drew upon new studies suggesting that proteins in vegetables like peanuts and soy could have a nutritional value (almost) equal to animal proteins.^111^ In 1955, concerned with the global ‘protein gap’, WHO and FAO, with the financial support of UNICEF and the Rockefeller Foundation, set up the Protein Advisory Group to promote and coordinate the development of new, safe protein-rich foods in collaboration with nutrition research centres across the world. Research focused on enriching flour with proteins extracted from fish and plants such as soybeans, oilseeds, cottonseeds and peanuts. Beyond technical problems, however, these alternatives proved either too expensive or impracticable for kwashiorkor prevention (for instance, because poor children did not have access to bread), or even dangerous to infant health.^112^

FORÉAMI, IRSAC and colonial health and agriculture services participated in this research, mainly by testing the acceptability and nutritional value of fish and peanut flour.^113^ In 1957–58, IRSAC researcher Demaeyer also studied the nutritional value and popular acceptance of protein-rich biscuits manufactured by the international food company Nestlé. However, despite Nestlé lobbying in Brussels, senior health and agricultural officers in the Colonial Ministry did not sign the contract Nestlé had in mind, arguing that this solution was too expensive and, with the biscuits being produced in Angola, against Belgium’s national interests.^114^ Most officials continued to consider local agricultural development (as described in the *Plan décennal* and the agreement with UNICEF/FAO/WHO) the most sustainable solution to protein malnutrition. FORÉAMI doctors, who had long collaborated with agricultural services to improve agricultural techniques and promote protein-rich crops such as peanuts, maize and sesame, now supported the expansion of fish-farming and the introduction of cattle in the Kwango sector.^115^ Surprisingly, an experiment by local veterinary services had shown that, in certain conditions, cattle could be raised even on the poor, sandy soils around Feshi.^116^

However, the UNICEF-sponsored milk campaign also had another afterlife. It set in motion a debate about the economic advantages of turning the Congolese into regular milk-consumers. In 1952, the Dutch origin of UNICEF milk had raised questions among doctors and nutritionists in Belgium, who viewed it as a missed opportunity for the dairy industry in Belgium. UNICEF’s policy was not to buy milk in the countries it assisted; and the Dutch had a flourishing milk exporting industry.^117^ Knowing that this restriction would no longer apply after the end of UNICEF’s engagement, officials in both the health and agricultural departments of the Colonial Ministry started inquiring whether, from 1955 onwards, the Belgium dairy industry could produce and ship the milk powder itself. Interest groups such as the National Milk Office and the Federation of Milk Producers alongside the Ministries of Economic Affairs and of Agriculture were adamantly positive. They emphasised that the Belgian milk industry overproduced skimmed milk powder (as a by-product of butter) that was hard to sell on a domestic market already saturated with fresh milk.^118^ Moreover, some dairies were already exporting high-quality skimmed milk powder to tropical countries such as India and Pakistan.^119^ They also pointed out that the settler dairy industry in Katanga and Ituri was not yet producing milk powder in sufficient quantity and quality.^120^

As a consequence, the government of the Belgian Congo bought Belgian skimmed milk powder for its anti-kwashiorkor campaign from 1955 onwards. Driven by the economic rationales of the metropole, the colonial government even considered extending its milk distribution programme. In 1955, the Belgian representative at the FAO, Philippe d’Otreppe, suggested that surplus Belgian milk powder could be used to improve nutritional conditions in the so-called *centres extra-coutumiers*, peri-urban areas for ‘detribalised’ African labourers and their families, where people had little access to proteins.^121^ In so doing, the government would follow recent FAO recommendations to increase the general use of milk products in low-consumption regions. Beyond improving diets, it would create a new and potentially burgeoning demand for milk products that could be met by the Belgian dairy industry.^122^ Neither the Colonial Ministry nor the Governor-General were against such an ‘economic turn’^123^ of the question and the colonial government set up a pilot programme distributing reconstituted milk to women, infants and children in the *centres extra-coutumiers* of Léopoldville and Matadi in the Lower Congo, a region where milk production was virtually non-existent.^124^ Henceforth, turning the Congolese into milk-consumers was no longer seen as a risk for the colonial budget, but an economic gain for the metropole.

## Conclusion: understanding international health in the Belgian Congo

During the last decades of colonial rule, Belgian colonial authorities, health agencies and researchers intensely engaged with kwashiorkor, a severe syndrome affecting mainly young children that was attributed chiefly to protein malnutrition. As it became a main concern of (colonial) governments and international organisations, the Belgian government in the early 1950s set up a joint campaign with UNICEF, FAO and WHO to fight kwashiorkor by distributing skimmed milk to ill people and rural populations at risk. The first of its kind in colonial Africa, the milk campaign met with some reservations on the part of Congolese women and Belgian (health) officials, but was particularly pushed by the health workers of the FORÉAMI in the south-western Kwango district and praised by the international health agencies.

Analysing how FORÉAMI and other actors viewed, implemented, reshaped, publicised and sustained the campaign not only adds to our knowledge of late-colonial nutritional policies, but also opens new perspectives on international health cooperation in late-colonial Africa. Here, my interpretation diverges from Jessica Pearson’s claims that, fearing UN interference in colonial affairs, French authorities only engaged with UN-sponsored health campaigns because they needed money.^125^ Certainly, the Belgians also feared international oversight and, as I have shown, strove to limit the influence of UN agencies by controlling how and to whom milk was distributed, and how, how often and by whom the programme was evaluated. Upon reading FORÉAMI’s first report, the Minister of Colonies clearly felt this had been successful, describing the campaign as a ‘good example of collaboration with an international agency in which we have ceded none of our rights and prerogatives’.^126^

Money, however, cannot have been the main motivation for the Belgian government. Not only did they not really need the external funding, as Dr Kivits emphasised in 1955, they knew from its conception that the campaign, given its division of labour and UNICEF rules about colonial co-funding, might be more expensive for the Belgian colonial state than for UNICEF.^127^ In the end, distributing the UNICEF milk cost about double ($430 000) from what UNICEF had paid to buy and ship the milk ($236 700). A large share of the Belgian expenses was incurred by FORÉAMI.^128^ Moreover, in 1953, the Belgian government promised UNICEF an allowance of $200 000 for its support in the Congo. Part of this sum would be paid by the colony: the government inscribed $25 000 in the annual budget of the Belgian Congo in 1955 and 1956.^129^ If the Belgian authorities primarily wanted money from UNICEF, they grossly miscalculated.

While money may have been an initial incentive, Belgian engagement with UNICEF and the other UN agencies was increasingly driven by the prospect of enhancing national prestige. The Belgian authorities used the campaign to show the world what they considered ‘a medical organisation that was, in many regards, at the avant-garde under the territories in Central Africa’, as a senior Belgian health official put it a few years later, when UNICEF was interested in sponsoring another campaign in the Belgian Congo.^130^ Arguably, this confidence built on the widespread idea among Belgian health officials that their health services and in particular FORÉAMI had a particularly strong record in preventative rural healthcare and infant welfare work. Being at the medical avant-garde was also an integral part of the self-image of FORÉAMI.^131^ FORÉAMI’s itinerant milk distribution scheme simultaneously capitalised on and showcased the organisation’s long-standing expertise in mobile and preventative rural healthcare. By quickly publishing a first report on the campaign, establishing a fully fledged nutritional laboratory to study its impact and experimenting with other protein-rich foodstuffs, FORÉAMI highlighted its scientific and practical know-how (and hence independence from international technical assistance) while also shaping international views of the campaign. Explaining why the programme was prolonged in early 1954, UNICEF’s programme director concluded that ‘FORÉAMI has played an important role in the execution of the programme and [that] most of the provisional conclusions that we were thus far able to draw were due to the mastery that it has shown’.^132^ FORÉAMI helped the Belgian government to fruitfully engage with international agencies and control the narrative on its colonial medicine.

## Footnotes

1 Algemeen Rijksarchief, Brussels (henceforth ARA), H 1531, Poudre de lait fournie, 2 September 1952; Ministère des Affaires Étrangères, Archives Africaines/Afrika-Archief, Brussels (henceforth MAEAA), AE 3005.1010, Recommendation (E/ICEF/L. 549), 12 February 1954.

2 On aetiology, see Jennifer Tappan , The Riddle of Malnutrition: The Long Arc of Biomedical and Public Health Interventions in Uganda (Athens: Ohio University Press, 2017), 1–5 ; John Nott, ‘“No one may starve in the British Empire”: Kwashiorkor, Protein and the Politics of Nutrition Between Britain and Africa’, *Social History of Medicine*, 34 (2021), 553–576: 561.

3 See Nick Cullather , The Hungry World: America’s Cold War Battle Against Poverty in Asia (Cambridge: Harvard University Press, 2010); Ruth Jachertz and Alexander Nützenadel, ‘Coping with Hunger? Visions of a Global Food System, 1930–1960’, *Journal of Global History*, 6 (2011), 99–119; Amanda Kay McVety, *The Rinderpest Campaigns: A Virus, Its Vaccines, and Global Development in the Twentieth Century* (Cambridge: Cambridge University Press, 2018).

4 The main exception was Kenneth J. Carpenter , Protein and Energy: A Study of Changing Ideas in Nutrition (Cambridge: Cambridge University Press, 1994), 142–79.

5 Tappan, *op. cit.* (note 2); Nott, *op. cit.* (note 2); Lola Wilhelm , ‘“One of the Most Urgent Problems to Solve”: Malnutrition, Trans-Imperial Nutrition Science, and Nestlé’s Medical Pursuits in Late Colonial Africa’, Journal of Imperial and Commonwealth History, 48 (2020), 914–33.

6 According to Tappan, it was only ‘by 1955’ that ‘the agenda shifted from short-term curative initiatives toward prevention’. See Tappan, *op. cit.* (note 2), 48.

7 See Randall M. Packard , A History of Global Health: Interventions into the Lives of Other Peoples (Baltimore: John Hopkins University Press, 2016), 7 (quote); James Webb and Tamara Giles-Vernick, ‘Introduction’, in Tamara Giles-Vernick and James Webb (eds), *Global Health in Africa: Historical Perspectives on Disease Control* (Athens: Ohio University Press, 2013), 1–21: 2–3.

8 For an historical overview, see Anne-Emmanuelle Birn , Yogan Pillay and Timothy H. Holtz (eds), Textbook of Global Health, 4th edn (Oxford: Oxford University Press, 2017), 1–88.

9 See Jessica Lynn Pearson , The Colonial Politics of Global Health: France and the United Nations in Postwar Africa (Cambridge: Harvard University Press, 2018); Philip. J. Havik, ‘Regional Cooperation and Health Diplomacy in Africa: From Intra-Colonial Exchanges to Multilateral Health Institutions’, *Manguinhos. História Ciências Saúde*, 27 (2020), 123–44; Philip J. Havik and José Pedro Monteiro, ‘Portugal, the World Health Organisation and the Regional Office for Africa: From Founding Member to Outcast (1948–1966)’, *Journal of Imperial and Commonwealth History*, (2021), https://doi.org/10.1080/03086534.2021.1892374. For inter-war health cooperation, see also Samuël Coghe, ‘Inter-imperial Learning and African Health Care in Portuguese Angola in the Interwar Period’, *Social History of Medicine*, 28 (2015), 134–54.

10 Compare to Pearson, *op. cit.* (note 9), 142–63.

11 On these two dimensions, see Nancy Rose Hunt , A Nervous State: Violence, Remedies, and Reverie in Colonial Congo (Durham: Duke University Press, 2016).

12 For infant healthcare, see Nancy Rose Hunt , ‘“Le Bébé en Brousse”: European Women, African Birth Spacing and Colonial Intervention in Breast Feeding in the Belgian Congo’, International Journal of African Historical Studies, 21 (1988), 401–3211617207; Idem, *A Colonial Lexicon of Birth Ritual, Medicalization, and Mobility in the Congo* (Durham: Duke University Press, 1999).

13 See Giovanni Trolli, *L’Assistance Médicale aux Indigènes du Congo Belge et Notre Dynastie: Historique et nouvelle méthode adoptée par FORÉAMI* (Anvers, 1935); Giovanni Trolli, ‘Méthode originale d’Assistance médicale aux indigènes en milieu rural appliquée au Congo belge: FORÉAMI’, *Bruxelles-Médical*, 20 (1939–40), 147–84; 217–53; 272–308; 324–48; R. Mouchet, ‘The FORÉAMI’, *Transactions of the Royal Society of Tropical Medicine and Hygiene*, 44 (1951), 483–500. On the links with Angola, see Coghe, *op. cit.* (note 9), 152–4.

14 Trolli, ‘Méthode originale’, *op. cit.* (note 13), 154–5; 239.

15 FORÉAMI (ed.), *Rapport annuel, 1938* (Bruxelles, 1939), 5–6; 80–3; 98–101; Trolli, ‘Méthode originale’, *op. cit.* (note 13), 165–8. See also F. Lamal , Essai d’étude démographique d’une population du Kwango: Les Basuku du Territoire de Feshi (Bruxelles: ARSOM, 1949).

16 For statistics, see E.M. Demaeyer , ‘Le problème du kwashiorkor au Congo belge’, Annales de la Société Belge de Médecine Tropicale, 38 (1958), 393–408: 394; 398.13606610

17 On early descriptions and aetiological uncertainty, see H.C. Trowell , J.N.P. Davies and R.F.A. Dean , Kwashiorkor (London: Arnold, 1954), 1–41 ; Tappan, *op. cit.* (note 2), 13–22.

18

Cicely Williams , ‘Kwashiorkor: A Nutritional Disease of Children Associated with a Maize Diet’, The Lancet, 226 (1935), 1151–2 (quote 1151). See also Jennifer Stanton, ‘Listening to the Ga: Cicely Williams’ Discovery of Kwashiorkor on the Gold Coast’, in Lawrence Conrad (ed.), *Women and Modern Medicine* (Amsterdam: Rodopi, 2001), 149–72.

19

Michael Worboys , ‘The Discovery of Colonial Malnutrition Between the Wars’, in David Arnold (ed.), Imperial Medicine and Indigenous Societies (Oxford: Oxford University Press, 1988), 208–225 ; Vincent Bonnecase, ‘When Numbers Represented Poverty: The Changing Meaning of the Food Ration in French Colonial Africa’, *Journal of African History*, 59 (2018), 463–81; Jonathan E. Robins, ‘“Food Comes First”: The Development of Colonial Nutritional Policy in Ghana, 1900–1950’, *Global Food History*, 4 (2018), 168–88.

20

John Boyd Orr and J.L. Gilks , The Physique and Health of Two African Tribes (London: H.M.S.O., 1931); Cynthia Brantley, ‘Kikuyu-Maasai Nutrition and Colonial Science: The Orr and Gilks Study in late 1920s Kenya revisited’, *International Journal of African Historical Studies*, 30 (1997), 49–86.

21 Samuël Coghe, *Extending the Pastoral Frontier: The Introduction of Cattle Farming in French Equatorial Africa during Late Colonialism* (Commodities of Empire Working Paper 28) (London, 2017).

22 Trowell, Davies and Dean, *op. cit.* (note 17), 12–41. On vitamins, see Carpenter, *op. cit.* (note 4), 119–41.

23 Tappan, *op. cit.* (note 2), 13–22.

24 Giovanni Trolli, *Résumé des observations réunies, au Kwango, au sujet de deux affections d’origine indéterminée* (Bruxelles: FORÉAMI, 1938), 37–57; G. van Daele, ‘Sur une affection de carence et de déséquilibre diététique observée au Congo (Buaki des indigènes)’, *Annales de la Société Belge de Médecine Tropicale*, (1938), 653–69. See also Trolli, ‘Méthode originale’, *op. cit.* (note 13), 165–8.

25 See G. Pieraerts , ‘Étude sur le syndrôme dépigmentation-oedème au Kasai’, Recueil de Travaux de Sciences Médicales au Congo Belge, 1 (1942), 104–24; C. Dricot, Beheyt and P. Charles, ‘Contribution à l’étude du Kwashiorkor (Mbuaki du Kwango)’, *Annales de la Société Belge de Médecine Tropicale*, 31 (1951), 581–630: 581–6.

26
*Ibid.*, 586.

27 See for instance Trowell, Davies and Dean, *op. cit.* (note 17), 1–11. For the Belgian Congo, see G. Doucet , ‘Le “Mbuaki” ou maladie de carence observée au Kwango’, Recueil de Travaux de Sciences Médicales au Congo Belge, 5 (1946), 261–73; Dricot, Beheyt and Charles, *op. cit.* (note 25).

28 Nott, *op. cit.* (note 2), 567–8.

29 See MAEAA, GG 18209, Dupuy, Note du Médecin-Directeur du FORÉAMI, 3 March 1937 and Dr. De Brauwere (Médecin-Directeur du FORÉAMI) to Governor of Province of Léopoldville, 28 June 1944; MAEAA, AE 3005.1010, A. Duren, Observations, 24 July 1951.

30 Trolli, *op. cit.* (note 24); Trolli, ‘Méthode originale’, *op. cit.* (note 13), 165–8. Compare to studies highlighting the role of colonialism in promoting hunger: Raymond R. Gervais, ‘Creating Hunger: Labor and Agricultural Policies in Southern Mosi, 1919–1940’, in Dennis D. Cordell and Joel W. Gregory (eds), African Population and Capitalism: Historical Perspectives (Boulder: Westview Press, 1987), 109–21; Allen. F. Isaacman, *Cotton is the Mother of Poverty: Peasants, Work, and Rural Struggle in Colonial Mozambique, 1938–1961* (Portsmouth: Heinemann, 1996), and for the Belgian Congo, Osumaka Likaka, *Rural Society and Cotton in Colonial Zaire* (Madison: University of Wisconsin Press, 1997).

31

Georges Nzongola-Ntalaja , The Congo from Leopold to Kabila. A People’s History (London/New York: Zed Books, 2002), 28–9. See also Judith A Byfield et al., (eds), *Africa and World War II* (New York: Cambridge University Press, 2015).

32 Compare Nott, *op. cit.* (note 2), 573–4 (quote 574) with Trowell, Davies and Dean, *op. cit.* (note 17), 13–46; 52–5; Marcel Autret and Moisés Behar, ‘Le syndrome de polycarence de l’enfance en Amérique Latine (Kwashiorkor)’, *Bulletin of the World Health Organization*, 11 (1954), 891–966; ARA, H 1127, FAO, La malnutrition protéique, 5 May 1954 (I/R/Presse 54/13).

33 For a broad panorama of UN-assisted supplementary feeding programmes across the globe, see United Nations, *Special Study on Social Conditions in Non-Self-Governing Territories: Summaries and Analyses of Information Transmitted to the Secretary-General during 1955* (New York: United Nations, 1956), 129–37.

34 See Autret and Behar, *op. cit.* (note 32), 956.

35 Bonnecase, *op. cit.* (note 19); Pearson, *op. cit.* (note 9), 154–63.

36 Frederick Cooper, ‘Modernizing Bureaucrats, Backward Africans, and the Development Concept’, in Frederick Cooper and Randall Packard (eds), International Development and the Social Sciences: Essays on the History and Politics of Knowledge (Berkeley: University of California Press, 1997), 64–92 ; Joseph Morgan Hodge, Gerald Hodl and Martina Kopf (eds), *Developing Africa: Concepts and Practices in Twentieth-Century Colonialism* (Manchester: Manchester University Press, 2014).

37 Maarten Langhendries and Reinout Vander Hulst, ‘Gezondheidszorg: Het paradepaard van de Belgische kolonisatie?’, in Idesbald Goddeeris , Amandine Lauro and Guy Vanthemsche (eds), Koloniaal Congo: Een geschiedenis in vragen (Antwerpen: Polis, 2020), 305–313: 310.

38 Ministère des Colonies de la Belgique, *Plan décennal pour le développement économique et social du Congo Belge* (Bruxelles: Visscher, 1949), 19–23; 37–61 and 368–438. On the development plan, see Guy Vanthemsche, *Genèse et portée du “Plan décennal” du Congo belge (1949–1959)* (Bruxelles: ARSOM, 1994). See also Ministère des Colonies de la Belgique, *Plan décennal pour le développement économique et social du Ruanda-Urundi* (Bruxelles: Visscher, 1951).

39 See MAEAA, FOR 4652.189 and, for the study, E-L. Adriaens , ‘Recherches sur l’alimentation des populations au Kwango’, Bulletin Agricole du Congo Belge, 42 (1951), 227–70 and 473–552.

40

Louis van den Berghe , ‘Le problème de la nutrition étudié par l’IRSAC’, Folia Scientifica Africae Centralis, 1 (1955), 5–9 . See also ARA, H 1485, IRSAC, Note, 12 January 1955.

41

Jacques Vuylsteke et al., ‘Nutrition’, in Pieter G. Janssens , Maurice Kivits and Jacques Vuylsteke (eds), Médecine et hygiène en Afrique Centrale de 1885 à nos jours, 2 vols, vol. 1 (Bruxelles: Fondation Roi Baudoin, 1992), 303–51: 320–2.

42 See particularly Hunt, ‘Bébé’, *op. cit.* (note 12); Hunt, ‘Noise over Camouflaged Polygamy, Colonial Morality Taxation, and a Woman-Naming Crisis in Belgian Africa’, *Journal of African History*, 32 (1991), 471–94; Hunt, *Colonial Lexicon, op. cit.* (note 12); Hunt, ‘Rewriting the Soul in a Flemish Congo’, *Past and Present*, 198 (2008), 185–215. For efforts in Portuguese and other colonies, see Samuël Coghe, *Population Politics in the Tropics: Demography, Health, and Transimperialism in Colonial Angola* (Cambridge: Cambridge University Press, forthcoming), Chapter 5.

43 Hunt, Bébé, *op. cit.* (note 12), 421–3 (here quotes).

44 See John Kent , The Internationalization of Colonialism: Britain, France, and Black Africa, 1939–1956 (Oxford: Clarendon Press, 1992), 263–85; Pearson, *op. cit.* (note 9), 67–88; Havik, *op. cit.* (note 9), 131–9.

45 See the conference proceedings: *Conférence interafricaine sur l’alimentation et la nutrition. Dschang-Cameroun, 3–9 octobre 1949* (Paris: La documentation française, 1949); Colonial Office (ed.), *Malnutrition in African mothers, infants and young children: Report of the second inter-African conference on nutrition held under the auspices of the Commission for Technical Co-Operation in Africa south of the Sahara (C.C.T.A.) at Fajara, Gambia, 19th–27th November, 1952* (London: H.M.S.O., 1954) and *Conferência inter-africana de nutrição: 3. sessão, Luanda, Outubro 1956. Communicações*, 2 vols (London: CCTA, 1957). For the smaller expert meetings, see ARA, H 1484–1494.

46 Kent, *op. cit.* (note 44), 263–7; Pearson, *op. cit.* (note 9), 67–88; Havik, *op. cit.* (note 9), 133.

47 Havik and Monteiro, *op. cit.* (note 9), 9–13.

48 Pearson, *op. cit.* (note 9), 157–8. On rinderpest, compare to McVety, *op. cit.* (note 3), 131–7; 148–9.

49 Jachertz and Nützenadel, *op. cit.* (note 3), 101–5.

50
*Ibid.*, 105–12. See also McVety, *op. cit.* (note 3), 86–163; James Gillespie, ‘International Organizations and the Problem of Child Health, 1945–1960’, *Dynamis*, 23 (2003), 115–42; Cullather, *op. cit.* (note 3).

51 See also Bonnecase, *op. cit.* (note 19), 474–5.

52 Joint FAO/WHO Expert Committee on Nutrition, *Report on the First Session, Geneva, 24–28 October 1949* (WHO Technical Report Series 16.2) (Geneva: WHO, 1950), 2.

53
*Ibid.*, 15. On this Joint Expert Committee, see also Tappan, *op. cit.* (note 2), 21–2; 43.

54 See *Conférence interafricaine*, *op. cit.* (note 45).

55 Joint FAO/WHO Expert Committee on Nutrition, *Report on the Second Session, Rome, 10–17 April 1951* (WHO Technical Report Series 44) (Geneva: WHO, 1951), 22–9; J. F. Brock and Marcel Autret , ‘Kwashiorkor in Africa’, Bulletin of the World Health Organization, 5 (1952), 1–71.14925815PMC2554082

56 Tappan, *op. cit.* (note 2), 43; Carpenter, *op. cit.* (note 4), 160.

57 Brock and Autret, *op. cit.* (note 55), 47–50.

58
*Ibid.*, particularly 51–64 (quote 61).

59
*Ibid.*, 61.

60 Joint FAO/WHO Expert Committee on Nutrition, *op. cit.* (note 55).

61 Gillespie, *op. cit.* (note 50); Birn, Pillay and Holtz (eds), *op. cit.* (note 8), 46; 56. See also Maggie Black , Children First: The Story of UNICEF, Past and Present (New York: Oxford University Press, 1996), 63–4; Silvia Inaudi, “Milk Is Life’: Nutritional Interventions and Child Welfare: The Italian Case and Post-War International Aid’, in David Gentilcore and Matthew Smith (eds), *Proteins, Pathologies and Politics: Dietary Innovation and Disease from the Nineteenth Century* (London: Bloomsbury Academic, 2019), 149–60.

62 UNICEF, *Report of the Executive Board, 22–24 May 1951 (E/2013)* (New York: United Nations, 1951), 7.

63 MAEAA, AE 3005.1010, Kivits, Note, 25 August 1955.

64 MAEAA, AE 3005.1010, A. Duren, Observations, 24 July 1951.

65 ARA, H 1527, Duren, Note pour Monsieur le Ministre, 18 October 1951; Henrard to Duren, 10 October 1951; Vanhove to Duren, October 1951.

66 Compare the slightly diverging figures in ARA, H 1527, Demande du Gouvernement, 24 October 1951 and ARA, H 1528, UNICEF, Recommendation (E/ICEF/R. 324), 5 April 1952, 7. The ‘orphan scheme’ was not directly related to kwashiorkor and will not be further discussed in this article.

67 ARA, H 1527, Simons to Duren, 5 October 1951 and Demande du Gouvernement, 24 October 1951; ARA, H 1528, UNICEF, Recommendation (E/ICEF/R. 324), 5 April 1952.

68 Pearson, *op. cit.* (note 9), 154–63.

69 ARA, H 1527, Vanhove to Duren, October 1951.

70 ARA, H 1528, Duren to Inspecteur Royal, 3 January 1952, Minister of Colonies to Minister of Foreign Affairs, 5 February 1952, and Minister of Foreign Affairs to Eggers (UNICEF), 25 March 1952.

71 See MAEAA, AE 3005.1010, Recommendation (E/ICEF/L. 549), 12 February 1954.

72 Compare ARA, H 1528, UNICEF, Recommendation of the Executive Director (E/ICEF/R. 324), 5 April 1952, 6–7 with C. Dricot and J. André, *Rapport du Fonds Reine Elisabeth sur l’action commune entreprise au Congo Belge par le gouvernement et le Fonds International de Secours à l’Enfance: Tome I: Campagne de distribution de lait écrémé* (Bruxelles: Imprimerie industrielle et financière, 1953), 6–7; 13; 15–9; ARA, H 1529, Rapport annuel distribution lait FISE dans la province de Kasai (1953), 20.

73 For details, see Dricot and André, *op. cit.* (note 72), 9–31.

74 Compare MAEAA, AGRI 211.59, Duren, Demande de lait (1952) with Hunt, Bébé, *op. cit.* (note 12), 422.

75 See Hunt, Bébé, *op. cit.* (note 12), 402–10. See also Coghe, *op. cit.* (note 42), Chapter 5.

76

A. Bal , ‘L’industrie laitière du C.B. et du Ruanda-Urundi’, Bulletin Agricole du Congo Belge, 42 (1951), 987–1006.

77 Similarly, Hunt, Bébé, *op. cit.* (note 12), 423–4; 428–9.

78 Christian Løvold Storhaug, Svein Kjetil Fosse and Lars T. Fadnes, ‘Country, Regional, and Global Estimates for Lactose Malabsorption in Adults: A Systematic Review and Meta-Analysis’, *Lancet Gastroenterology & Hepatology*, 2 (2017), 738–46. Both Hunt, Bébé, *op. cit.* (note 12), 428 and Tappan, *op. cit.* (note 2), 38 mention lactose intolerance.

79 See Dricot and André, *op. cit.* (note 72), 20–7; 45.

80
*Ibid.*, 56–7.

81
*Ibid.*, 41; Marcel Autret and R.C. Burgess, ‘Supplementary Feeding Programmes in Africa: The Example of the Belgian Congo’, in Colonial Office, *op. cit.* (note 45), 326–8.

82 Dricot and André, *op. cit.* (note 72), 31–51.

83

K. Holemans , Les carences alimentaires au Kwango (Bruxelles: Institut Royal Colonial Belge, 1954), 10.

84 Dricot and André, *op. cit.* (note 72), 52–74.

85
*Ibid.*, 74–82.

86
*Ibid.*, 85–6 (quote 86).

87 FORÉAMI (ed.), *Rapport sur l’activité durant l’année 1953* (Bruxelles, 1954), 127–9.

88 Holemans, *op. cit.* (note 83). See also Robert Ysebaert, ‘Étude statistique de l’influence de la distribution du lait FISE sur le poids et la taille des bénéficiaires Basuku du Cercle de Feshi en 1953’, in FORÉAMI (ed.), *Verslag over de werkzaamheden gedurende 1954* (Brussel, 1955), 200–3.

89 UNICEF Archives, New York (henceforth UA), PD-COR02–0096, M. Autret, R.C. Burgess and R. Marti, Rapport de la mission FAO/OMS/UNICEF sur le programme d’alimentation supplémentaire au Congo Belge et au Ruanda-Urundi, Octobre 1952 (FAO/53/2/877), 11 February 1953, 9–16; 27.

90 MAEAA, AE 3005.1010, Recommendation (E/ICEF/L. 549), 12 February 1954. Quote from UA, PD-COR02–0097, Marcel Autret, Note on the results of the supplementary feeding program in the Belgian Congo, September 1954, 9.

91 UNICEF, *Report of the Executive Board (22–24 April 1952)* (New York: United Nations, 1952). On protein deficiencies in the AEF, see also Coghe, *op. cit.* (note 21).

92 WHO Archives, Central Files (1946–1950), AS 8, Acting Director to V.A. Sutter, 27 November 1953; United Nations, *op. cit.* (note 33), 132–3. See also Autret and Burgess, *op. cit.* (note 81), 324 and Pearson, *op. cit.* (note 9), 161.

93 ARA, H 1529, Rapport annuel distribution lait FISE dans la province de Kasai, s.d.; Dr Leroy, Rapport de la mission médicale du Kwango, août 1952–août 1953, 26 February 1954 (quote); Dr Baudart, Rapport Campagne F.I.S.E. 1952–1953, 5 January 1954.

94 ARA, H 1120, Governor-General to Minister of Colonies, 13 April 1953.

95 See Maryinez Lyons , ‘The Power to Heal: African Auxiliaries in Colonial Belgian Congo and Uganda’, in Dagmar Engels and Shula Marks (eds), Contesting Colonial Hegemony: State and Society in Africa and India (London: I.B. Tauris & Co, 1994), 202–23; Samuël Coghe, ‘Disease Control and Public Health in Colonial Africa’, *Oxford Research Encyclopedia of African History*, (2020), 12–3. https://doi.org/10.1093/acrefore/9780190277734.013.620.

96 Compare FORÉAMI (ed.), *Rapport sur l’activité durant l’année 1952* (Bruxelles, 1953), 12 with Dricot and André, *op. cit.* (note 72), 44–6; 50–1.

97 ARA, H 1120, Governor-General to Minister of Colonies, 13 April 1953.

98 ARA, H 1120, Governor of Kasai Province to Governor-General, 6 March 1953.

99 Compare MAEAA, AGRI 211.59, Dr Duren, Note, 21 December 1954 and Governor-General to Minister of Colonies, 11 June 1955 with ARA, H 1120, Dr Thomas to Colonial Ministry, 26 July 1955.

100 FORÉAMI (ed.), *Rapport sur l’activité durant l’année 1957* (Bruxelles, 1958), 167. See also MAEAA, AGRI 211.59, Dr Thomas to Minister of Colonies, 11 September 1953.

101
*Ibid.*, 167.

102 Compare FORÉAMI, *op. cit.* (note 96), 55 with FORÉAMI, *op. cit.* (note 100), 134; 187.

103 Vuylsteke, *op. cit.* (note 41), 322.

104 FORÉAMI (ed.), *Rapport sur l’activité durant l’année 1956* (Bruxelles, 1957), 45; 99.

105 Dricot and André, *op. cit.* (note 72), 87–93.

106

K. Holemans , A. Lambrechts and H. Martin , Résultats d’une campagne d’alimentation supplémentaire par protéines végétales (arachides) (Bruxelles: Académie Royale des Sciences Coloniales, 1956).

107
*Ibid.*, 9–10.

108
*Ibid.*, 17–69.

109 FORÉAMI, *op. cit.* (note 104), 45.

110 Trowell, Davies and Dean, *op. cit.* (note 17), 195–202 (quote 198).

111
*Ibid.*

112 Carpenter, *op. cit.* (note 4), 153–79; Tappan, *op. cit.* (note 2), 47–51. See also Josh Ruxin , ‘The United Nations Protein Advisory Group’, in David F. Smith and Jim Phillips (eds), Food, Science, Policy and Regulation in the Twentieth Century: International and Comparative Perspectives (London: Routledge, 2000), 151–66.

113 See, for instance, M. Lassance , W. Bervoets and G. Evrard , ‘L’avenir des farines de poisson au Congo belge: Essais d’acceptabilité’, Annales de la Société Belge de Médecine Tropicale, 38 (1958), 669–78; E.M. Demaeyer and H. Vanderborght, ‘A Study of the Nutritive Value of Proteins from Different Sources in the Feeding of African Children’, *Journal of Nutrition*, 62 (1958), 335–52. See also the correspondence in MAEAA, AGRI 212 and ARA, H 1120.

114 See the correspondence (1957–1959) in MAEAA, AGRI 212.61. For a more positive account of Nestlés lobbying efforts in colonial Africa, see Wilhelm, *op. cit.* (note 5), 923–6.

115 FORÉAMI, *op. cit.* (note 100), 50. On fish farming, see United Nations, *op. cit.* (note 33), 134; M. Huet , Dix années de Pisciculture au Congo belge et au Ruanda-Urundi: Compte rendu de mission piscicole (Groenendaal-Hoeilaart: Ministère de l’Agriculture, 1957).

116

C. Miknevicius , ‘Élevage bovin au Kwango: Étude expérimentale de ses possibilités sur les hauts-plateaux sablonneux du système du Kalahari en Territoire de Feshi’, Bulletin Agricole du Congo Belge et du Ruanda-Urundi, 50 (1959), 1601–28.

117 ARA, H 1120, Duren to Minister of Colonies, 13 December 1952; MAEAA, AGRI 251.59, Lefebvre to Minister of Colonies, 3 September 1954. See also Dricot and André, *op. cit.* (note 72), 24.

118 MAEAA, AGRI 211.59, Minister of Colonies (Buisseret) to Governor-General, 9 July 1955; MAEAA, AGRI 211.59, Rey (Ministry of Economic Affairs) to Buisseret (Minister of Colonies), 3 September 1954.

119 MAEAA, AGRI 211.59, Lefebvre (Ministry of Agriculture) to Buisseret (Minister of Colonies), 3 September 1954.

120 See the debate in MAEAA, AGRI 211.59, Staner to Colonial Ministry, 27 March 1953; Staner to Governor-General, 3 August 1953 and ARA, H 1120, Minister of Colonies to Governor-General, 12 May 1953.

121 MAEAA, AGRI 211.59, D’Otreppe to Minister of Colonies, 11 February 1955. Grouping so-called ‘detribalised’ Africans, that is African migrants living beyond customary law, these centres had been officially sanctioned by Belgian law and given official statutes from the 1930s onwards, see Guy Baumer , Les centres indigènes extracoutumiers au Congo belge (Paris: Domat-Montchrestien, 1939).

122 MAEAA, AGRI 211.59, Cardon (FAO) to Belgian Ministry of Economic Affairs, 27 October 1954, enclosed in D’Otreppe to Minister of Colonies, 8 November 1954.

123 MAEAA, AGRI 211.59, Dr Guyaux (Veterinary Consultant in Colonial Ministry), Poudre de lait écrémé, s.d.

124 MAEAA, AGRI 211.59, Minister of Colonies (Buisseret) to Governor-General, 22 April 1955 and 9 July 1955; Governor-General to Minister of Colonies (Buisseret), 11 June 1955; Bertaux to d’Otreppe, 30 June 1956.

125 Pearson, *op. cit.* (note 9), 142–63.

126 Handwritten note (23 July 1953) on ARA, H 1529, Kivits to Minister of Colonies, 22 July 1953.

127 Pearson also draws attention to this, see Pearson, *op. cit.* (note 9), 150.

128 MAEAA, AE 3005.1010, Kivits, Note, 25 August 1955 and Recommendation (E/ICEF/L. 549), 12 February 1954, 6–7; 10–11; ARA, H 1529, Kivits to Minister of Colonies, 22 July 1953.

129 MAEAA, AE 3005.1010, UNICEF Belgium, Memorandum, June 1955 and further documents.

130 ARA, H 1526, Note sur la collaboration du Congo Belge et du RU avec l’OMS et le FISE, 1959.

131 See, for instance, Trolli, ‘Méthode originale’, *op. cit.* (note 13) and the discussion in Mouchet, *op. cit.* (note 13), 492–500. See more broadly also Hunt, *op. cit.* (note 11), 10.

132 MAEAA, AE 3005.1010, Recommendation (E/ICEF/L. 549), 12 February 1954, 3.

